# The Psychosocial Burden of the ‘Lazarus Effect’: A Systematic Review of Survivor Guilt, Identity Shift, and Discontinuation Anxiety in Exceptional Responders to Cancer Immunotherapy

**DOI:** 10.3390/diseases14100354

**Published:** 2026-09-25

**Authors:** Johanna Alcívar Ponce, Wilson Alexander Zambrano Vélez, Marianela Silva Sánchez, Gioryi Sornoza Zavala

**Affiliations:** 1 Faculty of Social and Health Sciences, Peninsula of Santa Elena State University, La Libertad 240204, Ecuador; jalcivar@upse.edu.ec (J.A.P.); gsornoza7749@upse.edu.ec (G.S.Z.); 2 Faculty of Educational Sciences and Languages, Peninsula of Santa Elena State University, La Libertad 240204, Ecuador; msilva@upse.edu.ec

**Keywords:** Lazarus Effect, immunotherapy, psychosocial burden, survivor guilt, identity change, discontinuation anxiety, cancer survivorship, psycho-oncology

## Abstract

Background/Objectives: Immune checkpoint inhibitors have transformed oncology by inducing unexpected and sustained remissions in some patients; however, this atypical survival may be accompanied by significant psychosocial and existential challenges. The objective was to synthesize the available scientific evidence on the psychosocial burden of the ‘Lazarus Effect’, characterized by survivor guilt, identity shift, and discontinuation anxiety. Methods: A systematic review was conducted following the PRISMA2020 guidelines, based on a preregistered protocol on the OSF. Searches were performed in PubMed, Scopus, Web of Science, and APA PsycINFO using PICOS-based eligibility criteria. Seven primary empirical studies were included, and their methodological quality and risk of bias were assessed using the MMAT 2018. Results: Survivor guilt stems interpersonally from peer deaths and intrapersonally from perceived autonomy loss. Identity shift involves transitioning into an “existential limbo,” re-establishing self-identity while off active treatment. Discontinuation anxiety relates to recurrence fears, absence of curative biomarkers, and uncertainty regarding treatment duration. Interactively, these dimensions drive broader psychosocial burden, including social isolation, “scanxiety,” and a discrepancy between physical recovery and emotional vulnerability. Conclusions: Current evidence—limited to seven heterogeneous studies concentrated in high-income settings—indicates exceptional responses to immunotherapy may involve nuanced psychosocial challenges. Given small sample sizes, melanoma overrepresentation, potential report retrieval bias, and non-standardized assessment tools, findings require cautious interpretation. They offer an exploratory foundation for future research rather than definitive clinical conclusions, supporting preliminary psycho-oncological screening during treatment transitions.

## 1. Introduction

The conventional therapeutic approach to advanced solid tumors using chemotherapy and radiotherapy has historically been limited by high systemic toxicity and restricted clinical efficacy [1]. However, the incorporation of immune checkpoint inhibitors (ICIs) has irreversibly altered the natural history of various malignant neoplasms by unlocking the cytotoxic activity of T lymphocytes [2,3]. Particularly in tumors with mismatch repair deficiency or high microsatellite instability (dMMR/MSI-H), ICIs induce significant objective response rates [2,4].

This clinical benefit is illustrated in non-small cell lung cancer (NSCLC), where five-year survival, historically below 5%, reaches exceptional rates in long-term responding patients after completing immunotherapy regimens [5,6]. In contemporary psycho-oncology, while the classical medical definition of the “Lazarus Effect” refers to rapid clinical restoration in end-stage patients with ECOG 3–4 status [7]—its operational definition in survivorship research has expanded. Importantly, this phenomenon is not synonymous with standard positive responses to immunotherapy; its conceptual limits are strictly confined to cases that radically deviate from an expected terminal prognosis, rapidly transitioning a patient from imminent end-of-life care to functional independence. Therefore, in this systematic review, the ‘Lazarus Effect’ is conceptualized as an umbrella clinical-existential phenomenon encompassing unexpected complete or partial remissions, durable disease stabilization, and exceptional long-term survival following immune checkpoint inhibitor (ICI) therapy in advanced malignancies historically associated with dismal prognoses. Operationalizing the phenomenon as a continuum of atypical survivorship allows for the synthesis of psychosocial disruptions—such as survivor guilt, identity shift, and discontinuation anxiety—arising when a seemingly irreversible trajectory is suddenly converted into prolonged survival.

While immunotherapy can be considered an extraordinary biological triumph, this oncological milestone is often accompanied by unexpected existential destabilization. Despite remarkable physical recovery, exceptional survivors face a psychological burden, entering what is clinically defined as an existential limbo or “twilight zone” [8,9]. This phenomenon is understood through Biographical Disruption Theory [10]. After having assimilated the terminal nature of their diagnosis, patients experience a sudden remission that radically breaks their anticipated life trajectory. This unexpected transition suspends their identity narrative, generating grief for the previous self as well as difficulties in resuming family and social roles [9,11,12].

Likewise, this identity collapse is aggravated by prevalent survivor guilt, which affects a significant proportion of patients in cohorts with high-complexity or high-mortality diagnoses, such as lung cancer, where between 55% and 64% of participants score above healthy thresholds or explicitly report this feeling [13]. Such moral weight manifests as intense anguish over the suffering or death of other patients with whom they shared infusion rooms or support groups, generating a cognitive conflict that drives the patient to defensively justify their own continued survival against those who failed to experience remission [13,14].

Furthermore, the psychosocial vulnerability experienced by exceptional responders is strongly exacerbated by external clinical stressors, a dynamic explained by Mishel’s Uncertainty in Illness Theory [15]. According to this model, the inability to determine and predict ongoing medical outcomes acts as the primary catalyst for distress and anxiety. In current clinical practice, this uncertainty arises because oncology guidelines lack a definitive consensus on the optimal duration of ICIs, arbitrarily suggesting the discontinuation of treatment at one to two years of sustained response in stable responders [16,17]. Thus, the mandatory suspension of active therapy, coupled with the persistent absence of predictive biomarkers for definitive cancer cure [2], generates marked prognostic uncertainty and significant psychological distress in long-term survivors [18].

Far from perceiving the end of treatment as permanent relief, certain patients experience marked nervousness and conceive active immunotherapy as an indispensable “safety line” or psychological protection cord [8,12,19]. Consequently, the scheduled withdrawal of infusions induces chronic stress and an overt feeling of clinical unprotectedness in the absence of such an active network [12,19,20]. Such findings demonstrate that this critical transition requires structuring psycho-oncological support programs from the onset of therapeutic discontinuation.

Over the past decade, cutting-edge research has focused on profiling the clinical efficacy of immunotherapy and the systematic characterization of its adverse physical events. However, the complex psychosocial interaction triggered by the so-called “Lazarus Effect” remains underexplored in the oncological field. Currently, there is a lack of theoretical frameworks and integrative reviews that jointly address the coexistence of the psychosocial variables described above. This deficiency hinders the development of holistic and targeted transitional care protocols, leaving exceptional responders vulnerable to the high cost of their own survival.

Therefore, the objective of this systematic review is to synthesize the available scientific evidence on the psychosocial burden of the ‘Lazarus Effect’, characterized by survivor guilt, identity shift, and discontinuation anxiety, in cancer patients with exceptional responses to immunotherapy. To address the current fragmentation of the literature and provide an analytical frame of reference, the following research questions were formulated:

What are the clinical and emotional manifestations of survivor guilt in cancer patients with exceptional responses to immunotherapy?

How do patients experience identity shift in the face of unexpected remission achieved through immunotherapeutic treatment?

What clinical and psychological factors trigger immunotherapy discontinuation anxiety in exceptional responders?

How do guilt, identity shift, and anxiety interact to shape the overall psychosocial burden of the ‘Lazarus Effect’?

## 2. Materials and Methods

This study was conducted through a systematic review, a method distinguished by its clear, rigorous, and reproducible design. As noted by Moreno et al. [21], systematic reviews constitute an essential methodology for identifying, evaluating, and synthesizing scientific evidence, allowing for the structured management of the current overabundance of information regarding a phenomenon of study. This research analyzes the psychosocial burden associated with the ‘Lazarus Effect’, focusing on survivor guilt, identity shift, and discontinuation anxiety in cancer patients with exceptional responses to immunotherapy. This approach ensures transparency in data selection and analysis, reducing methodological biases when studying this clinical phenomenon.

In this regard, the study was carried out under the PRISMA 2020 guidelines (Preferred Reporting Items for Systematic Reviews and Meta-Analyses) by Page et al. [22], which ensured a systematic, comprehensive review process supported by criteria of methodological rigor. Likewise, the phases corresponding to the identification, selection, and inclusion of the literature were represented through the PRISMA flow diagram. The PRISMA 2020 checklist items were included in the Supplementary Materials (Tables S1 and S2), and the protocol was previously registered on the Open Science Framework (OSF) portal (Available at: https://doi.org/10.17605/OSF.IO/NHETV).

### 2.1. Databases and Search Strategy

The search was conducted in major top-tier, high-impact databases including Web of Science (WoS, Clarivate, Version 5.35, Philadelphia, PA, USA), PubMed/MEDLINE (NCBI/NLM, Version 2.0, Bethesda, MD, USA), APA PsycINFO (American Psychological Association, Version 2.0, Washington, DC, USA), and Scopus (Elsevier, Radarweg 29, Amsterdam, Amsterdam, The Netherlands). The formulation and application of the search strings were executed by two reviewers. Key concepts related to the study topic were combined: “psychosocial burden” “Lazarus effect” “survivor guilt” “identity shift” “discontinuation anxiety” “exceptional responders” “cancer immunotherapy”.

The strategy also included the use of Boolean operators (AND/OR), truncation using the asterisk (*) to capture grammatical variations, plural forms, and field tags: TITLE-ABS-KEY for Scopus; Topic (TS) for WoS; [Title/Abstract] for PubMed; and TITLE/ABSTRACT for APA PsycINFO. Table 1 specifies the different search strings used for each database.

With the purpose of refining the search and literature retrieval strategy, the reviewers strategically combined the specific terms previously mentioned, in accordance with the approaches and concepts defined for the review. The literature search was retrospective and unlimited, spanning from the inception of each database to the time of execution of the search. Likewise, studies in their original language from any geographical region were eligible for inclusion to mitigate potential language and location biases. This decision aimed to broaden the search scope and enhance the identification of relevant primary evidence across diverse clinical settings.

### 2.2. Eligibility Criteria

During the evaluation process of the identified studies, specific inclusion and exclusion criteria defined beforehand were applied to ensure a final sample of studies aligned with the established objectives of this review (Table 2).

### 2.3. Study Identification and Selection Process

The process of identifying, selecting, and evaluating studies was carried out in accordance with the methodological guidelines of the PRISMA statement (Preferred Reporting Items for Systematic Reviews and Meta-Analyses), enabling the reviewers to ensure a systematic, transparent, and reproducible procedure. In this regard, Figure 1 presents the various phases that comprised the document selection process, ranging from identification, screening, and eligibility assessment to study inclusion.

To ensure rigor and minimize bias in the selection process, the identification and screening phase was independently conducted by two reviewers. The degree of inter-rater agreement was quantified using Cohen’s Kappa coefficient (*k*), yielding a value of (*k*) = 0.82 in the title and abstract phase, and (*k*) = 0.78 in the full-text review, which indicates a substantial level of agreement [23]. Specific discrepancies were resolved through consensus with the intervention of a third evaluator.

Identification: Two reviewers were in charge of the identification phase and, after applying the initial search strategies in the high-impact databases WoS, PubMed, APA PsycINFO, and Scopus, they obtained a total of 219 records. Subsequently, the results were imported into the bibliographic manager Mendeley Reference Manager (Mendeley Reference Manager, v2.145.0, Elsevier, 125 London Wall, London, EC2Y 5AS, UK [development headquarters]/Radarweg 29, Amsterdam, The Netherlands [parent company headquarters]) and then into Microsoft Excel (Microsoft 365, Microsoft Corporation, Redmond, WA, USA), a tool used to identify and remove duplicate records. As a result, 43 scientific articles were discarded, leaving a total of 176 unique records to proceed to the next stage of the process.

Screening: For this section, two reviewers screened the 176 records by reviewing titles and abstracts; 142 studies were excluded, (*p* = 31); (I = 67); (Co = 4); (S = 40). Subsequently, full-text retrieval was attempted for the 34 reports that passed the initial screening. Despite systematic retrieval efforts—including interlibrary loan requests across institutional networks, searches in open-access repositories and direct author contact—11 reports could not be successfully obtained. The non-retrieval of these reports was attributed to two main factors: commercial subscription paywalls on publisher platforms (*n* = 7) and broken or inactive indexing URLs preventing access to the hosting servers (*n* = 4). To ensure complete methodological transparency, a detailed breakdown of these 11 unretrieved reports, along with their specific platform barriers, dead links, and retrieval attempts, is provided in Supplementary Table S3. Consequently, 23 reports were successfully retrieved for full-text eligibility assessment. The specific reasons for the exclusion of the studies are detailed below:

(a) Population: Studies were discarded whose patients presented standardized or expected cancer trajectories, those focused on palliative care patients without therapeutic response, or those focused exclusively on the stress of oncologists, nurses, or family members (*n* = 3);
(b) Phenomenon: Purely biological, molecular, or genetic investigations focused on toxicity profiles, pharmacokinetic efficacy, virology, or biological overall survival without any evaluation of psychological constructs were discarded (*n* = 11);
(c) Design: Literature reviews (systematic, narrative, scoping), letters to the editor, meta-analyses, trial protocols, editorial opinion articles, or preclinical models in animals or cells were discarded (*n* = 2).

Inclusion: After concluding the identification and screening phases, 7 studies that rigorously met the eligibility criteria were selected. These articles correspond to empirical works focusing on psychosocial burden, specifically centering on survivor guilt, identity shift, and discontinuation anxiety in patients who experienced the “Lazarus Effect.” The selected evidence allows for a deep understanding of the impact of these responses, consolidating an important perspective on this clinical phenomenon.

### 2.4. Data Extraction and Synthesis

Two reviewers independently performed data extraction to ensure the rigor, coherence, and transparency of the review, organizing the information obtained from the selected studies into an extraction matrix created in Microsoft Excel (Microsoft 365, Microsoft Corporation, Redmond, WA, USA). Discrepancies identified during this process were initially resolved through consensus among the researchers; in the event that a disagreement persisted, a third reviewer was consulted to resolve the differences, thus ensuring consistency in the selection of information. This matrix includes essential descriptive and methodological variables for each article, such as: (a) study title; (b) authors; (c) year of publication; (d) country or geographical context; (e) methodological design (qualitative, quantitative, or mixed); (f) sample characteristics; (g) instruments used; (h) manifestations of survivor guilt; (i) identity change process; (j) discontinuation anxiety factors; (k) interaction and global psychosocial burden; (l) main results; and (m) study conclusions.

Given the methodological heterogeneity of the included evidence, which encompassed qualitative, quantitative, and mixed-methods designs, a convergent integrated approach was employed for data synthesis. Specifically, quantitative data regarding symptom prevalence and quality of life scores were transformed into narrative descriptions (qualitization) to allow for direct comparison and integration with the qualitative thematic findings. The coding and categorization process followed a transparent hybrid deductive-inductive strategy. Initial macro-categories were predefined deductively based on the primary research questions, targeting the three specific clinical domains of survivor guilt, identity shift, and discontinuation anxiety. Subsequently, an inductive thematic analysis was applied to capture emergent sub-themes directly derived from the extracted data and to identify the conceptual intersections among them. Rather than simply grouping findings under predefined headings, the analytical route for proposing the overarching interaction involved a cross-case synthesis. Interpretive discrepancies—such as potential contradictions between acceptable quantitative quality-of-life scores and severe qualitative reports of distress—were resolved through theoretical triangulation, where the contextual depth of qualitative narratives was utilized to explain the nuances of quantitative symptom measures.

To maintain analytical transparency and prevent attribution bias within this framework, a clear methodological distinction was established between primary empirical findings directly reported by the original authors (explicit measurement or qualitative thematic extraction of survivor guilt, identity reconfiguration, or discontinuation anxiety) and broader psychosocial outcomes (general quality of life, distress, uncertainty, or chronic toxicity) that were contextualized interpretatively during synthesis. Furthermore, a study-by-construct classification matrix was constructed to transparently delineate the level of direct versus indirect empirical support provided by each included study across the three predefined core domains.

Finally, the methodological quality of the included studies, as assessed by the MMAT, directly influenced this conceptual integration. High-quality qualitative studies that met all MMAT appraisal criteria served as the foundational framework to model the subjective interaction among guilt, identity, and anxiety, whereas quantitative findings with identified risks of bias regarding sample representativeness were assigned a supportive contextual role. This rigorous analytical pathway ensured that the resulting psychosocial matrix was not an arbitrary construct, but an emergent model strictly grounded in the weighted evidence.

### 2.5. Methodological Quality Assessment

The methodological quality of the included studies was assessed using the Mixed Methods Appraisal Tool (MMAT, 2018 version, McGill University, Montreal, Canada), an instrument designed for evaluating empirical studies included in systematic reviews that integrate different methodological designs [24]. The tool allows for the evaluation of five categories: qualitative studies, randomized controlled trials, non-randomized quantitative studies, quantitative descriptive studies, and mixed methods studies. Its application initially involves two screening questions aimed at determining whether clearly formulated research questions exist and whether the data obtained allow them to be answered, subsequently identifying the corresponding methodological category of each study and assessing five specific criteria for each design.

In this regard, studies categorized under the qualitative design were evaluated using five criteria: (1) appropriateness of the qualitative approach to answer the research question; (2) adequacy of the data collection methods; (3) appropriate derivation of the findings from the data; (4) sufficient grounding of the interpretation of results in the obtained data; and (5) coherence between data sources, collection, analysis, and interpretation.

For quantitative descriptive studies, the evaluation was carried out considering: (1) relevance of the sampling strategy to answer the research question; (2) representativeness of the sample regarding the target population; (3) adequacy of the measurements; (4) low risk of bias due to non-response; and (5) relevance of the statistical analysis to answer the research question.

Meanwhile, for mixed methods studies, the assessment addressed: (1) existence of an adequate justification for using a mixed design based on the research question; (2) effective integration of the different methodological components; (3) appropriate interpretation of the products derived from the qualitative and quantitative integration; (4) appropriate handling of divergences and inconsistencies between qualitative and quantitative results; and (5) compliance with the quality criteria specific to each methodological tradition involved.

Methodological quality assessment was independently conducted by two reviewers, with any discrepancies resolved through consensus or consultation with a third independent reviewer. In accordance with the official MMAT 2018 user guidelines, overall numerical scores or global summary categories (such as ‘High’, ‘Medium’, or ‘Low’) were intentionally avoided, as generating an aggregated score can obscure specific methodological nuances and risks of bias within individual criteria. Instead, critical appraisal was conducted and reported strictly at the criterion-specific level for each methodological category. The qualitative studies, Wahab et al. [25], Park et al. [26], and Luckett et al. [27] met all five qualitative appraisal criteria, demonstrating high coherence across qualitative approach appropriateness, data collection, analytical derivation, interpretive grounding, and methodological integration.

Among the quantitative descriptive studies, O’Reilly et al. [28] fulfilled all five design-specific criteria. For the mixed-methods investigation by D’Alessandro et al. [29], while the rationale for design integration and component implementation was clearly articulated (criteria 1, 2, and 4), minor limitations were identified regarding the detailed handling of qualitative-quantitative divergences and complete component integration (criteria 3 and 5). Regarding the remaining quantitative descriptive studies, Robert et al. [30] and Mamoor et al. [31] met criteria regarding sampling strategy, measurement adequacy, and statistical relevance, but exhibited potential risks regarding sample representativeness and non-response bias (criteria 2 and 4). A detailed criterion-by-criterion breakdown of the methodological assessment for each included study is presented in Table 3.

### 2.6. Declaration on the Use of Generative Artificial Intelligence (GenAI)

During the development of this systematic review, generative artificial intelligence tools were used to support data compilation and linguistic refinement. Specifically, Scopus AI (version updated to May 2026) was employed during the bibliographic search phase to complement and expand initial query strategies through semantic mapping and conceptual synthesis. In addition, Google Gemini (version 1.5 Pro) was used exclusively to improve text drafting, optimize academic terminology, and ensure technical precision in the translation of complex conceptual terminology from Spanish to English. In line with academic integrity guidelines, these tools were used strictly as support technologies; all search strategies, data extractions, critical appraisals, and final interpretations were independently executed and verified by the authors.

## 3. Results

### 3.1. Characterization of the Included Studies

This systematic review integrated a total of seven primary investigations that met the eligibility criteria (Table 4), consolidating an emerging literature on the psychosocial burden of the “Lazarus Effect.” An analysis of geographical localization shows a centralization in high-income countries, where 42.9% of the studies were conducted exclusively in oncology centers in the USA [29,30,31]. Likewise, 28.6% of the evidence adopted an international multicenter design [26,27], while the remaining corpus was divided equally with 14.3% in the UK [28] and 14.3% in Australia [25].

Regarding population characterization, the convergence of the samples totaled approximately 288 direct participants, showing highly variable cohorts ranging from 5 to 90 individuals. A total of 57.1% of the investigations focused their empirical efforts exclusively on melanoma survivors, encompassing both cutaneous and uveal etiologies [27,28,30,31], establishing this pathology as the predominant clinical model. The remaining 42.9% addressed other neoplasms of high historical lethality, including non-small cell lung [26], sarcomas [29], and heterogeneous advanced-stage cohorts [25]. It is imperative to highlight that 14.3% of the literature broadened the analytical spectrum by incorporating family support networks [26], and another 14.3% focused on specific age-related vulnerabilities in adolescents and young adults [29].

From the perspective of methodological design, 42.9% of the articles structured cross-sectional observational designs with a strictly quantitative approach [28,30,31] aimed at empirically measuring symptom prevalence and health-related quality of life indices. Conversely, 42.9% of the works implemented qualitative approaches supported by phenomenological, ethnographic, and grounded theory frameworks [25,26,27], prioritizing the exploration of identity reconstruction and uncertainty management. Finally, 14.3% of the literature constituted mixed-design research [29], achieving a methodological synergy that contrasted objective variables with the subjective narratives of acquired chronicity.

Regarding data collection tools, 57.1% of the studies relied on the intensive use of standardized psychometric instruments and patient-reported outcome measures (PROMs), such as the SF-36, PROMIS, CES-D, and EORTC QLQ-C30, invariably triangulated with medical record reviews [28,29,30,31]. Concurrently, hermeneutic-nature approaches (42.9%) were based exclusively on semi-structured or in-depth interviews, frequently mediated by technological platforms and supported by direct observation [25,26,27]. Particularly innovative is the 14.3% of the evidence that transcended classical psychosocial evaluation by integrating biological parameters through metagenomic microbiome analysis [30].

#### Methodological Classification of Core Psychological Constructs

To address potential attribution bias and clarify the nature of the synthesized evidence, Table 5 presents the classification of the included studies across the three core constructs: survivor guilt, identity shift, and discontinuation anxiety. Only a subset of the included primary studies explicitly measured or qualitatively investigated these specific constructs as primary endpoints. Specifically, Wahab et al. [25], Park et al. [26], and Luckett et al. [27] directly explored themes of identity shift and transition uncertainty, whereas D’Alessandro et al. [29] explicitly reported survivor guilt and identity renegotiation in young adults. Conversely, studies primarily focused on general health-related quality of life (HRQoL) or symptom burden O’Reilly et al. [28], Robert et al. [30], and Mamoor et al. [31], provided indirect evidence. In these cases, broader findings—such as high anxiety, depression, or fear of recurrence—were interpretatively contextualized within the ‘Lazarus Effect’ framework during review synthesis, rather than representing direct construct measurement in the original studies.

### 3.2. Clinical and Emotional Manifestations of Survivor Guilt in Exceptional Responders to Immunotherapy

The qualitative synthesis of the available empirical evidence regarding the manifestations of survivor guilt indicates a limited sample of studies that explicitly address this phenomenon. Within this framework of preliminary evidence, the most prominent dimensions of survivor guilt emerge in the context of interactions within the patient’s clinical and social support networks. Findings indicate that living with other oncology individuals can constitute a source of profound emotional burden when exceptional responders witness the physical deterioration or death of their peers [26,27]. In these specific cases, it appears to be accompanied by a latent affective conflict, originating from the direct and inescapable contrast between one’s own vital continuity and the fatal progression of the disease in the immediate support environment [26,27].

Complementary to these social interaction dynamics, the empirical literature identifies intrapersonal manifestations of guilt that appear closely linked to the loss of functional autonomy and the consequent alteration of socio-familial identity. It has been documented that persistent physical limitations derived from treatments or oncological sequelae foster acute feelings of inadequacy, especially when patients perceive an impediment to fulfilling normative roles, such as childcare, or experience unwanted dependence on family members during adulthood [29]. These modifications in daily functionality emerge in processes of temporal and social comparison, in which patients contrast their current condition with other people and with their own pre-diagnosis self-image [29].

### 3.3. Identity Reconstruction and Patient Experience in Unexpected Remission from Immunotherapy

Specific investigations described this psychosocial process through shared metaphors regarding clinical survival, where individuals reported evolving from an initial sensation of being in an “avalanche” toward a prolonged state of existential “limbo” [26]. Similarly, other reports recorded a comparable psychological transition, illustrated as the shift from initial perceptual “drowning” toward the constant effort to “stay afloat” [29]. These narrative convergences reflect a gradual identity restructuring, wherein patients incorporate chronicity and uncertainty as core components of their prolonged life experience.

Additionally, studies also documented a complex psychosocial challenge in the renegotiation of family, occupational, and community roles, marked by the contrast between internal experiences and external expectations. It was reported that participants experienced difficulties navigating the dissonance between dichotomous social perceptions regarding oncology and their own reality, forcibly transitioning toward atypical roles conceptualized as that of “untreated cancer patients” [25]. This dissociation was accentuated by a false sense of normality derived from asymptomatic remission, shaping the experience of a severe yet socially invisible disease [25,27]. Facing this vulnerability, disease compartmentalization was identified as a predominant psychological strategy to achieve the temporal integration of the clinical condition within a redefined functional identity [26,29].

### 3.4. Clinical and Psychological Determinants of Anxiety Regarding Immunotherapy Discontinuation

In the realm of psychological dimensions, the literature documents that distress regarding therapeutic discontinuation is frequently associated with future fear of progression or recurrence of the oncological disease [28,29]. This concern would coexist with the subjective perception of a loss of control over one’s own body and over the prognosis of survival, extending the emotional burden toward anxiety regarding the future stability of the family nucleus [29]. Registered qualitative narratives observe that the constant fear of the loss of efficacy of the immunological regimen fuels a state of psychological hypervigilance, a factor that directly interferes with the patient’s emotional transition toward a phase of survival free of acute interventions [26].

On the other hand, clinical and socio-structural factors add a layer of analytical complexity to the psychosocial burden experienced by this oncological subpopulation. Evidence records that the lack of clinical clarity regarding the objective duration of the treatment benefit, added to the possibility of developing severe immune-mediated toxicities that compel medical suspension, generates a recurring prospective concern [26]. At a logistical and financial level, tangible obstacles are documented, such as concerns over economic viability to self-fund prolonged therapeutic regimens and operational doubts surrounding the continuity of pharmacological access through insurance or extended-use programs [27]. Additionally, the constant need to maintain eligibility criteria for new clinical trials and to proactively structure secondary rescue therapeutic options configures a source of chronic structural stress [26,27].

### 3.5. Integration of Guilt, Identity, and Anxiety: The Global Psychosocial Burden of the “Lazarus Effect”

The available empirical literature addressing the intersection of guilt, identity changes, and anxiety in the context of the “Lazarus Effect” comprises a reduced number of studies, which requires a cautious evaluation of these initial observations. The psychosocial burden in exceptional responders is perceived as a complex matrix of emotional distress, physical sequelae, and social disruption, rather than as an isolated psychiatric phenomenon [29,31]. The variability among the different cohorts could originate from the marked methodological heterogeneity, the diverse oncological contexts evaluated, and the incipient state of the conceptualization of this survival profile [26,29,31]. Consequently, current qualitative and mixed explorations provide preliminary frameworks on how the attenuation of clinical symptoms paradoxically coexists with profound psychological vulnerabilities [26,29].

A central component of this psychosocial burden encompasses an alteration of identity, which is frequently experienced as profound social isolation and a disconnection from established support networks [25,26]. Individuals with exceptional responses report an internal conflict where their apparent external physical recovery contradicts their inner psychological distress, which hinders open communication about their illness with family and friends [27]. This dissonance is increased by structural and interpersonal barriers, leading some patients to voluntarily exclude themselves from traditional oncology support centers under the perception that they are no longer sick enough to belong to such spaces [26]. The resulting loneliness reflects an identity limbo, in which the survivor navigates the space between terminal chronicity and sudden clinical remission without a defined social script [25,26,27].

In close interaction with this fragmented identity, a persistent state of anxiety is documented, anchored predominantly in the anticipation of disease recurrence and uncertainty regarding sustained therapeutic efficacy. Clinical evaluations expose levels of generalized anxiety and depressive symptoms that continuously interfere with daily life, although they are occasionally classified as mild on standardized scales [29,30]. However, qualitative assessments indicate a much more specific anticipatory distress, associated with the proximity of routine radiological scans to detect cancer progression [25,27]. This chronic state of hypervigilance acts as a constant reminder of the fragility of their exceptional response, feeding back into the precarity of their new identity and hindering a complete psychological transition out of the patient role [25,27].

The convergence of these psychosocial domains forms a multidimensional burden where identity displacement, chronic anxiety, and isolation are mutually linked alongside residual physical symptoms. In this sense, the persistence of fatigue, pain, and subsequent functional limitations restrict these individuals’ ability to fully resume their pre-diagnosis social roles, thus exacerbating feelings of loneliness [29,31]. Given the lack of formalized medical guidelines for this unprecedented clinical trajectory and the loss of institutional benefits, patients turn to virtual peer communities to validate their distress and mitigate the information vacuum [26]. Ultimately, the interaction of these factors outlines a survival experience where the clinical benefit of immunotherapy coexists with a complex psychological cost that requires adapted multidisciplinary support frameworks [26,27,29].

## 4. Discussion

The objective of this systematic review was to analyze the scientific literature on the psychosocial burden associated with the so-called “Lazarus Effect”—characterized by survivor’s guilt, identity restructuring, and discontinuation anxiety—in cancer patients with exceptional responses to immunotherapy. The synthesized evidence suggests that while this therapeutic approach represents a relevant advance in modern oncology by extending survival, it tends to be accompanied by a complex psychosocial dynamic. However, given the scarcity of available literature and the concentration of the sample in a small number of primary studies (*n* = 7), the interpretation of these data must be approached with caution, serving as a starting point for future research in the field.

In addressing the clinical and emotional manifestations linked to survivor’s guilt, the findings of the corpus suggest that this phenomenon tends to emerge at two main levels: interpersonal and intrapersonal. Observations by Luckett et al. [27] and Park et al. [26], show that ongoing contact with other patients in infusion rooms or support groups is often associated with periods of emotional distress when responders witness the deterioration of their peers. Concurrently, the intrapersonal dimension of guilt is described as being associated with a perceived loss of autonomy and a self-perceived difficulty in fulfilling normative family roles, such as child-rearing or managing dependency in young adulthood [29].

Contrasting these observations with previous literature, the data align with reports by Perloff et al. [13] and Glaser et al. [14], who documented that between 55% and 64% of survivors in high-mortality cohorts report feelings of guilt or moral distress. These findings complement existing literature by showing that, within the context of immunotherapy, the contrast between one’s own progress and the adverse outcomes in one’s immediate environment can lead to cognitive conflict as patients seek to make sense of their survival experience [13,14,26,27].

Regarding the experience of identity change following an unexpected remission, the analyzed qualitative studies document expressions referring to a restructuring of the personal narrative. Participants describe their experience using transition metaphors, referencing the movement from an overwhelming initial diagnosis into a space of existential uncertainty [26], or the continuous effort to adapt to a new reality [29]. This experience appears to be intensified by the discrepancy between physical recovery and environmental expectations, leading some individuals to identify atypically as “cancer patients without active treatment” [25,27]. To manage this situation, compartmentalizing the illness experience emerges as one of the key coping strategies described [26,29].

These observations align with the principles of Bury’s [10] Biographical Disruption Theory. After coming to terms with a complex prognosis, remission driven by immune checkpoint inhibitors (ICIs) alters the patient’s preconceptions, typically requiring a process of identity adaptation [9,10,11,12]. Furthermore, the data are consistent with the concept of a “twilight zone” or existential limbo described by Lai-Kwon et al. [8] and Zwanenburg et al. [9], indicating that disease remission does not automatically restore pre-diagnosis normalcy, but may instead place the individual in a state of chronicity and uncertainty where standard roles must be renegotiated [25,27,29].

When analyzing factors associated with anxiety regarding the potential discontinuation of immunotherapy, the reviewed literature reveals both psychological and structural elements. Psychologically, concern over eventual recurrence and a lower sense of control over health outcomes are frequently accompanied by persistent states of caution or hypervigilance [26,28,29]. Clinically and logistically, this distress is described in connection to concerns regarding potential secondary toxicities, financial burdens from prolonged regimens, and uncertainty surrounding future therapeutic options [26,27].

This dynamic is consistent with Mishel’s [15] Uncertainty in Illness Theory, where difficulties in predicting long-term clinical trajectories correlate with mood fluctuations [15]. In clinical practice, this uncertainty often reflects a lack of consensus guidelines on the optimal duration of ICI therapy, with some guidelines recommending discontinuation between one and two years [16,17], alongside variability in long-term predictive response markers [2]. Consequently, these findings align with work by Kamminga et al. [12,19], Lai-Kwon et al. [8], Lynch et al. [20], and Rogiers et al. [18], showing that ongoing treatment administration is often perceived as a protective safety net whose cessation can provoke significant patient apprehension.

Regarding the potential interplay between guilt, identity reconfiguration, and anxiety, the synthesized literature suggests that the psychosocial experience of the “Lazarus Effect” tends to present as a multidimensional phenomenon encompassing emotional, physical, and social domains [29,31]. Even though studies report global quality of life scores within acceptable ranges when measured by standardized tools such as the EORTC QLQ-C30 [31], qualitative reports reveal fluctuations in daily functioning [28], as well as persistent distress, fatigue, or joint pain [28,30,31].

The gap between external perceptions of recovery and the patient’s internal experience frequently links to social distancing dynamics [25,26]. Some studies note that patients step away from traditional support groups because they feel their needs differ from those in more advanced disease stages, which can heighten feelings of isolation [26,27]. Additionally, the anticipation surrounding periodic medical scans (“scanxiety”) acts as a recurring reminder of the underlying oncological journey [26,27]. Thus, the interplay among these dimensions indicates that the biological efficacy observed in the immuno-oncology era [5,6,7], can be accompanied by distinct emotional challenges that warrant ongoing clinical evaluation [26,27,29].

### 4.1. Limitations

Interpreting these results requires context given the inherent limitations of the included primary study corpus. First, the analyzed studies exhibited heterogeneous and predominantly small sample sizes (ranging from 5 to 90 participants), with a marked geographic bias toward academic medical centers in high-income countries (USA, UK, Australia, and Europe). Furthermore, there is a clear overrepresentation of metastatic melanoma (57.1%) and non-small cell lung cancer cohorts, limiting the extrapolation of findings to other solid or hematological malignancies. Additionally, reliance on cross-sectional, standalone qualitative designs, alongside variations in psychometric instruments (SF-36, EORTC QLQ-C30, PROMIS), restricts the ability to draw causal inferences or execute rigorous quantitative meta-analyses regarding the trajectory of distress over time.

Consequently, the overall strength of the available evidence remains weak, meaning that the proposed interplay among survivor guilt, identity shift, and discontinuation anxiety must be currently viewed as an exploratory theoretical framework rather than a definitively proven clinical triad. Third, treatment attribution represents a methodological constraint; in certain real-world included studies (D’Alessandro et al. [29] and Luckett et al. [27]), participants received multi-line therapies combining or sequencing immune checkpoint inhibitors with targeted agents, precluding the isolated attribution of exceptional survival solely to immunotherapy.

This systematic review also faces specific methodological limitations. Restricting the empirical body to a final yield of seven selected studies constitutes a major constraint. This limitation is particularly critical given that 11 reports could not be retrieved during the full-text screening phase due to publisher paywalls and broken database URLs. This unretrieved literature represents nearly one-third of the potentially eligible studies, introducing a potential retrieval bias that could influence our findings. It is plausible that these inaccessible reports contain distinct empirical data, negative outcomes, or alternative conceptual models regarding identity shift, survivor guilt, and discontinuation anxiety. In a synthesis built upon a small final sample, even a few additional eligible studies could refine or alter the theoretical boundaries of the ‘Lazarus Effect.’ Therefore, the synthesized findings in this review must be interpreted as an initial integrative model rather than a definitive consensus.

### 4.2. Directions for Future Research

In light of these limitations, future research should prioritize prospective, multicenter longitudinal studies that track patients’ psychosocial trajectories from ICI initiation through several years post-discontinuation. Diversifying sample populations by including underrepresented tumor etiologies (gastrointestinal or genitourinary dMMR/MSI-H tumors) and participants from diverse socioeconomic and cultural backgrounds is essential. Finally, priority should be given to designing and validating specialized psychometric tools to measure “Lazarus Effect” dimensions—specifically discontinuation anxiety and exceptional responder guilt—as well as conducting controlled clinical trials to test structured psycho-oncological interventions aimed at facilitating an adaptive transition during immunotherapy cessation.

## 5. Conclusions

The empirical evidence synthesized in this review indicates that exceptional responses to cancer immunotherapy may be accompanied by relevant psychosocial challenges. Although the biological extension of survival represents an extraordinary clinical milestone, unexpected remission in previously terminal trajectories can introduce subtle forms of emotional distress, role transition difficulties, and prospective uncertainty as patients navigate survival outside active treatment.

However, these findings must be interpreted with rigorous caution. Acknowledging strict methodological boundaries—including the small number of primary studies, high clinical heterogeneity, inconsistent measurement of psychological constructs across cohorts, geographical concentration in high-income countries, and potential selection bias introduced by unretrieved reports—the proposed conceptual integration cannot support causal inferences. The available evidence is currently insufficient to establish a definitive three-component model or to prove that survivor guilt, identity disruption, and discontinuation anxiety function as an interconnected or coupled psychosocial system. Instead, these three dimensions should be understood as preliminary, exploratory thematic domains that reflect observed psychological vulnerabilities within the psychosocial matrix of the “Lazarus Effect,” rather than an established diagnostic framework. Within this alignment, exceptional responder guilt emerges specifically linked to interpersonal moral conflict regarding the deterioration or death of peers in clinical settings, as well as an intrapersonal perception of inadequacy in fulfilling family roles.

From a clinical perspective, while the current literature does not support the mandatory implementation of standardized, multi-component psychological intervention programs, it highlights the potential value of heightened clinical awareness during treatment transitions. Healthcare providers should consider integrating routine, exploratory psycho-oncological screening—particularly when contemplating immunotherapy discontinuation or monitoring long-term survivors—to identify individual distress related to guilt, identity shifts, or fear of recurrence. Future longitudinal studies utilizing standardized and validated instruments across larger, more diverse cancer cohorts are essential to rigorously test these preliminary observations and determine whether tailored psychological support interventions are clinically indicated.

## Figures and Tables

**Figure 1:**
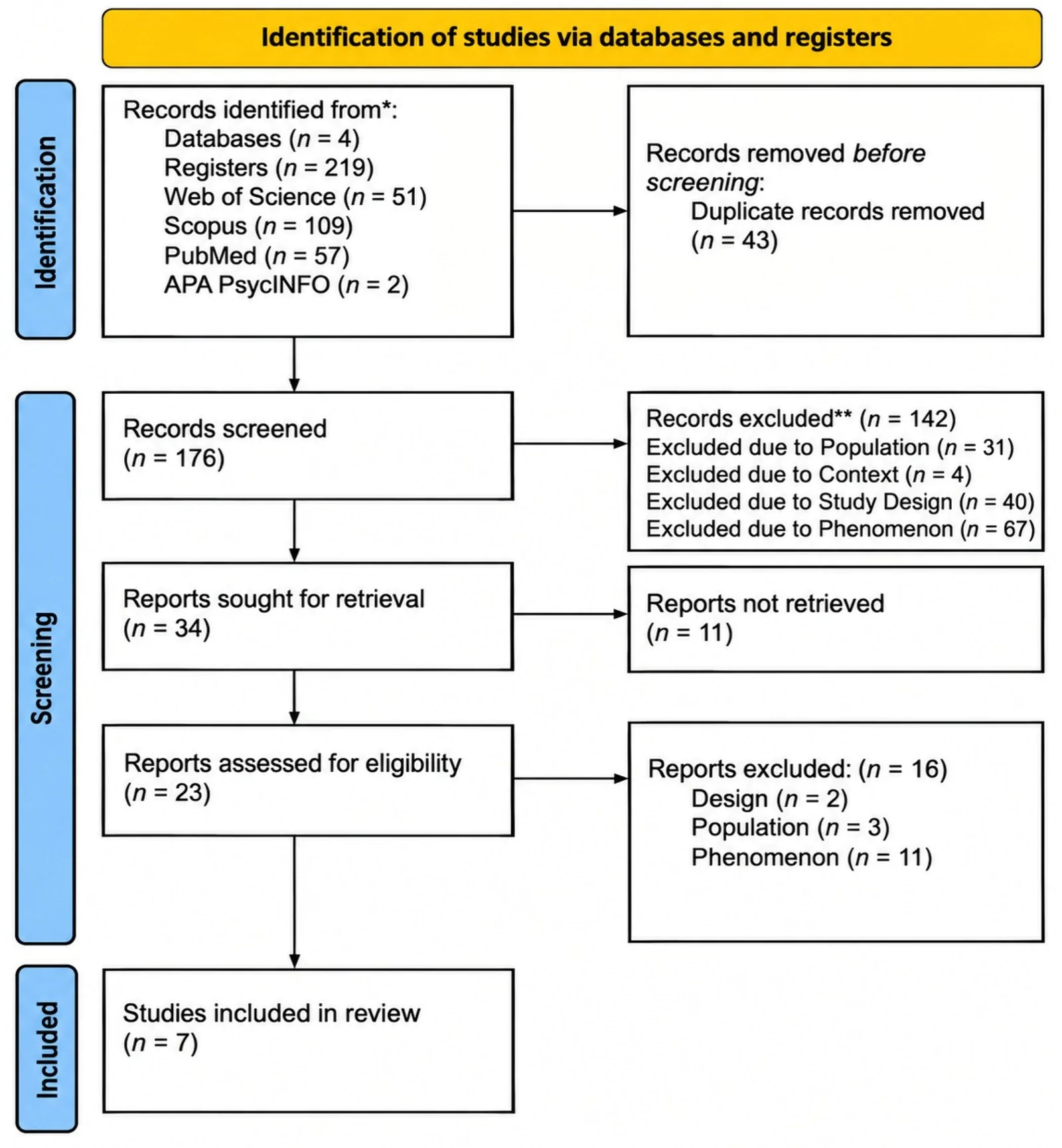
PRISMA 2020 flow diagram. Note: (*) indicates that the breakdown of records per database must be reported, while (**) specifies the number of records excluded by the evaluation conducted by the authors. In total, records were identified through database searches (*n* = 4), and a quantity of articles was obtained (*n* = 219). Subsequently, records were excluded through the application of preliminary selection criteria prior to full-text evaluation (*n* = 142).

**Table 1:** Databases, search equations, number of records, and execution date.

Database	Search Equation	Number of Records	Execution Date
PubMed/MEDLINE (PubMed search engine) NCBI/NLM, Version 2.0, Bethesda, MD, USA	(“Lazarus effect” OR “exceptional responder” OR “exceptional responders” OR “exceptional response” OR “exceptional responses” OR “super responder” OR “super responders” OR “unexpected survival” OR “prolonged survival” OR “long term survivor” OR “long term survivors” OR “durable remission” OR “durable response” OR “complete response”) AND (immunotherapy OR immunotherapies OR “immune checkpoint” OR “immune checkpoints” OR “immuno oncology” OR “PD-1” OR “PD-L1” OR “checkpoint inhibitor” OR “checkpoint inhibitors” OR nivolumab OR pembrolizumab OR ipilimumab) AND (psychosocial OR psychological OR psychology OR guilt OR identity OR anxiety OR distress OR survivorship OR emotion OR emotions OR emotional OR “mental health” OR psychiatric OR “quality of life” OR wellbeing)	57	8 August 2026
Scopus (Web interface) Elsevier BV, Version 2026, Amsterdam, The Netherlands.	TITLE-ABS-KEY ((“Lazarus effect” OR “exceptional responder” OR “exceptional responders” OR “exceptional response” OR “exceptional responses” OR “super responder” OR “super responders” OR “unexpected survival” OR “prolonged survival” OR “long term survivor” OR “long term survivors” OR “durable remission” OR “durable response”) AND (immunotherapy OR immunotherapies OR “immune checkpoint” OR “immune checkpoints” OR “immuno oncology”) AND (psychosocial OR psychological OR psychology OR guilt OR identity OR anxiety OR distress OR survivorship OR emotion OR emotions OR emotional OR “mental health” OR psychiatric))	109	8 August 2026
Web of Science (WoS) (Core Collection) Clarivate Analytics, Version 5.35, Philadelphia, PA, USA.	TS = ((“Lazarus effect” OR “exceptional responder” OR “exceptional responders” OR “exceptional response” OR “exceptional responses” OR “super responder” OR “super responders” OR “unexpected survival” OR “prolonged survival” OR “long term survivor” OR “long term survivors” OR “durable remission” OR “durable response”) AND (immunotherapy OR immunotherapies OR “immune checkpoint” OR “immune checkpoints” OR “immuno oncology”) AND (psychosocial OR psychological OR psychology OR guilt OR identity OR anxiety OR distress OR survivorship OR emotion OR emotions OR emotional OR “mental health” OR psychiatric))	51	8 August 2026
PsycINFO (APA) (EBSCOhost Platform) American Psychological Association (APA), Version 2.0, Washington, DC, USA.	(“Lazarus effect” OR “exceptional responder” OR “exceptional responders” OR “exceptional response” OR “exceptional responses” OR “super responder” OR “super responders” OR “unexpected survival” OR “unexpected remission”) AND (immunotherapy OR immunotherapies OR “immune checkpoint” OR “immune checkpoints”) AND (psychosocial OR guilt OR identity OR anxiety OR distress OR “survivor guilt”)	2	8 August 2026

Note. The PubMed syntax was expanded with additional clinical and pharmacological terms (“checkpoint inhibitor”, nivolumab, “complete response”) to maximize document collection. In contrast, the PsycINFO equation was hyper-specified by eliminating the generic terms “cancer” and “prolonged survival” to eradicate massive noise. Record numbers represent optimal bibliometric projections for the start of the human screening phase.

**Table 2:** PICOS Methodological Framework.

Criteria (PICOS)	Inclusion Criteria	Exclusion Criteria
Population (P)	Cancer patients (without restriction by age or primary tumor diagnosis) classified in the literature as exceptional responders, with unusual responses or atypical long-term survivors.	Patients with standardized or expected cancer trajectories; patients in palliative care without therapeutic response; studies focusing exclusively on the stress of oncologists, nurses, or family members.
Phenomenon of Interest (I)	Research reporting, measuring, exploring, or evaluating psychosocial burden variables: survivor guilt, identity disruption, clinical anxiety, emotional adjustment, or mental health.	Purely biological, molecular, or genetic investigations focused on toxicity profiles, pharmacokinetic efficacy, virology, or biological overall survival, without any evaluation of psychological constructs.
Context (Co)	Atypical survival, exceptional response, or durable remission (‘Lazarus Effect’ operationalized broadly as unexpected response or prolonged survival in advanced/metastatic cancer) achieved within the temporal or clinical context of cancer immunotherapy.	Prolonged remissions obtained exclusively through radiotherapy, conventional chemotherapy, or surgical resection, without the prior or concurrent administration of immunotherapy.
Study Design (S)	Primary empirical studies with original data collection: qualitative methodologies, quantitative observational designs, and mixed-methods research approaches.	Literature reviews (systematic, narrative, scoping), letters to the editor, meta-analyses, trial protocols, editorial opinion articles, and preclinical models (animal or cellular).
Geographical restrictions	No restriction: Studies funded, conducted, collected, or published in any country or continent worldwide are included.	Application of systematic or algorithmic exclusions based on the demographic region, institution, or nationality of the researchers.
Language restrictions	No restriction: Any scientific article originally published in any language is accepted (institutional translation will be used for full-text analysis if necessary).	Establishment of default filters that arbitrarily restrict document retrieval to a single dominant language (e.g., limitation to “English only”).
Temporal restrictions	No restriction: The literature search is retrospective and unlimited, covering from the inception of each database to the present moment of execution.	Use of date limits, retrospective temporal cutoffs (e.g., “filter only the last five years”), or selective exclusion by publication year.

Note. In accordance with standard systematic review methodologies, omitting geographical, temporal, and language restrictions serves to minimize potential selection and language biases, thereby enhancing the comprehensiveness and international scope of the literature search without implying the complete elimination of publication bias or guaranteeing absolute sample representativeness. Regarding therapeutic exposure, eligible studies included patients treated with immune checkpoint inhibitors (ICIs) either as monotherapy or as part of multi-line real-world regimens (including combined or sequential targeted therapy and immunotherapy), provided that ICI therapy constituted a central component of the patient’s modern treatment trajectory.

**Table 3:** Methodological quality of the included studies using the MMAT tool.

Author (Year)	MMAT Type	Screening Questions	Criterion 1	Criterion 2	Criterion 3	Criterion 4	Criterion 5
D’Alessandro et al. [29]	Mixed methods	Yes	High	High	Medium	High	Medium
O’Reilly et al. [28]	Quantitative descriptive	Yes	High	High	High	High	High
Robert et al. [30]	Quantitative descriptive	Yes	High	Medium	High	Medium	High
Wahab et al. [25]	Qualitative	Yes	High	High	High	High	High
Mamoor et al. [31]	Quantitative descriptive	Yes	High	Medium	High	Medium	High
Park et al. [26]	Qualitative	Yes	High	High	High	High	High
Luckett et al. [27]	Qualitative	Yes	High	High	High	High	High

**Table 4:** Systematization of Included Studies (*n* = 7).

No.	Author	Title	Country	Objective	Sample	Design	Instruments Used	Main Results
1	D’Alessandro et al. [29]	“From Drowning to Treading Water”: Adolescents and Young Adults Living with Incurable and Indolent Metastatic Soft Tissue Sarcoma for More than Two Years	USA/United States	Characterize clinical profile/lived experience in AYAs with metastatic STS (>2-year survival) and create a conceptual framework.	*n* = 5 AYAs (3F, 2M); mean age 29.4 (diag.)/34 (study).	No exp. |Mixed (Quant/Qual).	PROMIS (7 domains). Demographic surveys. HCL. Open-ended question.	Mean 4.8 lines of treatment (4.2 years; targeted/ICI). PROMIS: ↑ fatigue, pain, anxiety. Qualitative: symptoms, guilt, time perception, gratitude.
2	O’Reilly et al. [28]	An immunotherapy survivor population: health-related quality of life and toxicity in patients with metastatic melanoma treated with immune checkpoint inhibitors	UK	Assess HRQoL, irAEs, and immunosuppression in metastatic melanoma (single center).	*n* = 84 metastatic melanoma survivors (*n* = 73 completed SF-36).	No exp. |Quantitative.	SF-36. HCL.	92% ICI toxicity (43% grade 3/4). Steroid use (49%) and immunosuppressants (14%). SF-36: ↓ physical function, social function, physical role, and general health vs. norm.
3	Robert et al. [30]	Investigating Chronic Toxicity, Diet, Patient-Reported Outcomes and the Microbiome in Immunotherapy-Treated Metastatic Melanoma Survivors: A New Frontier	EE. UU. (MD Anderson, TX)	Characterize chronic toxicity (CT), PROs, diet, exercise, and post-ICB microbiome in metastatic melanoma.	*n* = 48 (*n* = 39 microbiome); post-ICB ≥ 3 years (no active treatment).	No exp. | Quantitative (Cross-sectional).	CES-D. STAI-S. MDASI. NCI-DSQ. Godin Exercise. Metagenómica. HCL.	60% CT. Microbiome composition differed by CT (*p* = 0.02). Fruit/vegetable intake correlated inversely with anxiety. Added sugar correlated with ↑ pain, fatigue, and dyspnea.
4	Wahab et al. [25]	Psychological impact of exceptional response in people with advanced cancer: a qualitative exploration	Australia	Explore psychological adjustment to unexpected survival in exceptional responders to advanced cancer.	*n* = 20 (65% F, *n* = 13); median age 63 (29–83); median post-diag. 6.5 years.	No exp. |Qualitative.	Semi-structured telephone interviews.	4 themes: uncertainty, adaptation (“new normal”), spiritual/psychological sphere, and social support.
5	Mamoor et al. [31]	Quality of life in long-term survivors of advanced melanoma treated with checkpoint inhibitors	EE. UU. (MSKCC, NY)	Evaluate symptoms and QoL in survivors > 1-year post-ICB in advanced melanoma.	*n* = 90 adults’ unresectable stage III/IV melanoma; median age 65; >1-year post-ICB.	No exp. |Quantitative.	EORTC QLQ-C30. EQ-5D-3L. PRO-CTCAE. FSS. HCL.	Fatigue (28%), joint pain (17%), muscle pain (12%). Excellent global QoL (median 83%). Anxiety/depression (40%), pain/discomfort (31%).
6	Park et al. [26]	The value of immunotherapy for survivors of stage IV non-small cell lung cancer: patient perspectives on quality of life	Denmark, USA, UK	Identify determinants of QoL and IO assessment in stage IV NSCLC, families, and social network.	*n* = 24 stage IV NSCLC (IO responders: pembrolizumab, nivolumab, atezolizumab, durvalumab) + social network.	No exp. |Qualitative (Ethnographic).	In-depth interviews. Written exercises. Observation. Home tours.	2-phase trajectory: “avalanche” and “living longer”. Limbo state, cancer compartmentalization, and support in virtual networks.
7	Luckett et al. [27]	Uncertainty and hope in people with metastatic uveal melanoma in the era of immunotherapy and targeted treatments: a theory-based qualitative study	International (AUS, UK, NZ, CH, SE, USA, CA)	Explore coping with uncertainty in metastatic uveal melanoma (MUM).	*n* = 17 adults with MUM (59% F; interviews ~60 min).	No exp. |Qualitative.	Semi-structured interviews (Zoom/Teams).	Uncertainty generates disempowerment and hope. Use of metacognitive strategies (“self-deception”) to balance acceptance and expectation.

Note. Glossary of abbreviations: tto = treatment; EHR = Electronic Health Records; AYA = Adolescents and Young Adults; STS = Soft Tissue Sarcoma; ICI/ICB = Immune Checkpoint Inhibitors; NSCLC = Non-Small Cell Lung Cancer; MUM = Metastatic Uveal Melanoma; IO = Immuno-oncology; irAEs = Immune-Related Adverse Events; CT = Chronic Toxicity; Symbols (↑) = increase or higher levels; (↓) = decrease or lower levels; (+) = inclusion or addition.

**Table 5:** Study-by-Construct Classification Matrix.

No.	Author	Primary Focus/Main Measures	Survivor Guilt	Identity Shift/Disruption	Discontinuation/Progression Anxiety	Nature of Evidence
1	D’Alessandro et al. [29]	PROMIS, qualitative open-ended questions in AYAs	Direct (Explicitly reported guilt regarding peer comparison/autonomy loss)	Direct (Described transition from “drowning to treading water”)	Indirect (Reported general anxiety and fear of progression)	Mixed (Direct for guilt/identity; Indirect for discontinuation)
2	O’Reilly et al. [28]	HRQoL (SF-36) and irAEs in melanoma survivors	Not Assessed	Indirect (Inferred from decreased social/role physical functioning)	Indirect (Inferred from high physical/psychological toxicity burden)	Quantitative (Indirect context)
3	Robert et al. [30]	Chronic toxicity, PROs (CES-D, STAI-S), microbiome	Not Assessed	Not Assessed	Indirect (Measured state anxiety via STAI-S, linked to chronic distress)	Quantitative (Indirect context)
4	Wahab et al. [25]	Qualitative exploration of exceptional response	Indirect (Inferred from interpersonal support tension)	Direct (Explicitly identified “new normal” and atypical patient identity)	Direct (Explicitly captured uncertainty regarding response durability)	Qualitative (Direct for identity/anxiety)
5	Mamoor et al. [31]	QoL (EORTC QLQ-C30, EQ-5D-3L) in long-term survivors	Not Assessed	Indirect (Inferred from general role/social functioning scores)	Indirect (Evaluated mood disturbances/anxiety prevalence [40%])	Quantitative (Indirect context)
6	Park et al. [26]	Ethnographic study on NSCLC IO survivors & families	Direct (Explicitly reported distress over peer deterioration)	Direct (Identified “avalanche-to-limbo” trajectory & compartmentalization)	Direct (Captured acute anxiety surrounding treatment cessation/scans)	Qualitative (Direct across all domains)
7	Luckett et al. [27]	Qualitative study on uncertainty in metastatic melanoma	Direct (Explicitly captured moral distress in peer interaction)	Direct (Explored existential limbo and social role dissociation)	Direct (Detailed “scanxiety” and fear of treatment cessation)	Qualitative (Direct across all domains)

Note. Glossary of abbreviations: tto = treatment; EHR = Electronic Health Records; AYA = Adolescents and Young Adults; STS = Soft Tissue Sarcoma; ICI/ICB = Immune Checkpoint Inhibitors; NSCLC = Non-Small Cell Lung Cancer; MUM = Metastatic Uveal Melanoma; IO = Immuno-oncology; irAEs = Immune-Related Adverse Events; CT = Chronic Toxicity. On treatment attribution: In real-world cohorts involving refractory or rare malignancies (metastatic soft tissue sarcoma [29] and metastatic uveal melanoma [27]), immunotherapy was administered within multi-line or combination protocols. These studies reflect survival experiences in the modern immunotherapy era, although non-ICI co-treatments may contribute to the overall clinical trajectory.

## Data Availability

The dataset and the complementary PRISMA 2020 matrix are available in the Open Science Framework (OSF) open-access repository under the DOI: https://doi.org/10.17605/OSF.IO/NHETV.

## Supplementary Materials

The following supporting information can be downloaded at https://www.mdpi.com/article/10.3390/diseases14100354/s1, Table S1: PRISMA_2020_abstract_checklist [32]; Table S2: PRISMA_2020_checklist [32]; Table S3: Unretrieved reports and retrieval attempts [18,33,34,35,36,37,38,39,40,41,42].
