# Supplementary material for: The Psychosocial Burden of the ‘Lazarus Effect’: A Systematic Review of Survivor Guilt, Identity Shift, and Discontinuation Anxiety in Exceptional Responders to Cancer Immunotherapy
Source: Diseases. 2026 Sep 25;14(10):354. doi: 10.3390/diseases14100354 (PMC13703520; doi:10.3390/diseases14100354)
Supplement: Supplementary file 1 [file diseases-14-00354-s001.zip › Table_S2_PRISMA_2020_checklist.pdf]

PRISMA

PRISMA 2020 Checklist

Section and Topic	Item #	Checklist item	Location where item is reported
**TITLE**			
Title	1	Identify the report as a systematic review.	Title, Page 1<br>Identifies as a systematic review: *"The psychosocial burden of the 'Lazarus Effect': A systematic review of survivor guilt, identity shift, and discontinuation anxiety in exceptional responders to cancer immunotherapy"*.
**ABSTRACT**			
Abstract	2	See the PRISMA 2020 for Abstracts checklist.	Abstract, Page 1<br>Formatted structured abstract (Background/Objectives, Methods, Results, Conclusions, Keywords).
**INTRODUCTION**			
Rationale	3	Describe the rationale for the review in the context of existing knowledge.	Introduction, Pages 1–3<br>Explains that while ICIs have transformed oncology, the complex psychosocial interaction triggered by the "Lazarus Effect" remains underexplored, lacking integrative frameworks.
Objectives	4	Provide an explicit statement of the objective(s) or question(s) the review addresses.	Introduction, Page 3<br>Stated at the end of Section 1: *"The objective of this systematic review is to synthesize the available scientific evidence on the psychosocial burden..."* along with 4 specific research questions.
**METHODS**			
Eligibility criteria	5	Specify the inclusion and exclusion criteria for the review and how studies were grouped for the syntheses.	Materials and Methods, Section 2.2, Pages 5–6<br>Detailed in Table 2 using the PICOS framework (Population, Phenomenon of Interest, Context, Study Design, plus geographical, language, and temporal criteria).
Information sources	6	Specify all databases, registers, websites, organisations, reference lists and other sources searched or consulted to identify studies. Specify the date when each source was last searched or consulted.	Materials and Methods, Section 2.1 & Table 1, Pages 3–5<br>Searched PubMed, Scopus, Web of Science, and APA PsycINFO on August 8, 2026. OSF repository consulted for protocol.
Search strategy	7	Present the full search strategies for all databases, registers and websites, including any filters and limits used.	Materials and Methods, Section 2.1 & Table 1, Pages 3–5<br>Full search equations with Boolean operators, truncations, and field tags for each database are specified in Table 1.
Selection process	8	Specify the methods used to decide whether a study met the inclusion criteria of the review, including how many reviewers screened each record and each report retrieved, whether they worked independently, and if applicable, details of automation tools used in the process.	Materials and Methods, Section 2.3, Pages 5–6<br>Two reviewers independently screened titles/abstracts and full texts. Inter-rater agreement calculated via Cohen's Kappa (k = 0.82 and k = 0.78). Mendeley and Excel used for deduplication.
Data collection process	9	Specify the methods used to collect data from reports, including how many reviewers collected data from each report, whether they worked independently, any processes for obtaining or confirming data from study investigators, and if applicable, details of automation tools used in the process.	Materials and Methods, Section 2.4, Page 7<br>Two reviewers independently extracted data into a structured Excel matrix; persistent discrepancies were resolved by a third reviewer.
Data items	10a	List and define all outcomes for which data were sought. Specify whether all results that were compatible with each outcome domain in each study were sought (e.g. for all measures, time points, analyses), and if not, the methods used to decide which results to collect.	Materials and Methods, Section 2.2 & 2.4, Pages 5, 7<br>Defined primary outcomes around psychosocial burden variables: survivor guilt, identity shift/disruption, clinical anxiety/discontinuation anxiety, and quality of life.

Section and Topic	Item #	Checklist item	Location where item is reported
	10b	List and define all other variables for which data were sought (e.g. participant and intervention characteristics, funding sources). Describe any assumptions made about any missing or unclear information.	Materials and Methods, Section 2.4, Page 7<br><br>Extracted title, authors, year, country, methodological design, sample size/characteristics, instruments used, and main conclusions.
Study risk of bias assessment	11	Specify the methods used to assess risk of bias in the included studies, including details of the tool(s) used, how many reviewers assessed each study and whether they worked independently, and if applicable, details of automation tools used in the process.	Materials and Methods, Section 2.5 & Table 3, Pages 7–9<br><br>Methodological quality assessed using MMAT 2018 independently by two reviewers, with a third arbiter for consensus.
Effect measures	12	Specify for each outcome the effect measure(s) (e.g. risk ratio, mean difference) used in the synthesis or presentation of results.	**N/A / No reportado.** Due to high study heterogeneity (qualitative/descriptive), no comparative effect measures (e.g., RR, OR, MD) or quantitative meta-analyses were executed.
Synthesis methods	13a	Describe the processes used to decide which studies were eligible for each synthesis (e.g. tabulating the study intervention characteristics and comparing against the planned groups for each synthesis (item #5)).	Materials and Methods, Section 2.4, Page 7<br><br>Eligible studies were grouped into thematic synthesis axes aligned with research questions based on reported qualitative narratives and quantitative scores.
	13b	Describe any methods required to prepare the data for presentation or synthesis, such as handling of missing summary statistics, or data conversions.	**No reportado.** Data transformation or statistical conversions were not conducted due to narrative synthesis approach.
	13c	Describe any methods used to tabulate or visually display results of individual studies and syntheses.	Materials and Methods, Section 2.4 & Table 4, Pages 7, 10–12<br><br>Tabulated using a comprehensive matrix (Table 4) detailing sample, design, tools, and main findings, accompanied by narrative text synthesis.
	13d	Describe any methods used to synthesize results and provide a rationale for the choice(s). If meta-analysis was performed, describe the model(s), method(s) to identify the presence and extent of statistical heterogeneity, and software package(s) used.	Materials and Methods, Section 2.4, Page 7<br><br>Narrative comparative synthesis was performed due to qualitative and cross-sectional methodological heterogeneity. Meta-analysis was not conducted.
	13e	Describe any methods used to explore possible causes of heterogeneity among study results (e.g. subgroup analysis, meta-regression).	**N/A.** Heterogeneity was explored qualitatively across tumor types, designs, and instruments; quantitative subgroup analysis/meta-regression was not performed.
	13f	Describe any sensitivity analyses conducted to assess robustness of the synthesized results.	**N/A.** Quantitative sensitivity analysis was not applicable to this qualitative and descriptive systematic review.
Reporting bias assessment	14	Describe any methods used to assess risk of bias due to missing results in a synthesis (arising from reporting biases).	Materials and Methods, Section 2.2, Page 5<br><br>Handled at design level by eliminating language, geographic, and temporal restrictions to mitigate publication bias. Formal statistical tests (e.g., funnel plots) were N/A.
Certainty assessment	15	Describe any methods used to assess certainty (or confidence) in the body of evidence for an outcome.	Materials and Methods, Section 2.5, Pages 7–9<br><br>Assessed via MMAT 2018 criteria grading overall evidence quality (High/Medium/Low) per methodological design. GRADE system was not applied.
**RESULTS**			
Study selection	16a	Describe the results of the search and selection process, from the number of records identified in the search to the number of studies included in the review, ideally using a flow diagram.	Results, Section 3.1 & Figure 1, Pages 5–6, 9<br><br>Reported in PRISMA flow diagram (Figure 1): 219 records identified, 43 duplicates removed, 176 screened, 142 excluded, 23 full texts assessed, 7 studies included.
	16b	Cite studies that might appear to meet the inclusion criteria, but which were excluded, and explain why they were excluded.	Materials and Methods, Section 2.3,

PRISMA 2020 Checklist

Section and Topic	Item #	Checklist item	Location where item is reported
			Pages 6–7<br>Detailed reasons for full-text exclusion (n=16) categorized by Population (n=3), Phenomenon (n=11), and Design (n=2). Specific citations of excluded studies are not listed.
Study characteristics	17	Cite each included study and present its characteristics.	Results, Section 3.1 & Table 4, Pages 9–12<br>All 7 included primary studies are cited ([25–31]) and systematically characterized in Table 4.
Risk of bias in studies	18	Present assessments of risk of bias for each included study.	Materials and Methods, Table 3, Pages 8–9<br>Summarized in Table 3: 4 studies rated "High" quality and 3 rated "Medium" quality across MMAT 2018 criteria.
Results of individual studies	19	For all outcomes, present, for each study: (a) summary statistics for each group (where appropriate) and (b) an effect estimate and its precision (e.g. confidence/credible interval), ideally using structured tables or plots.	Results, Table 4 & Sections 3.2–3.5, Pages 10–14<br>Individual study findings (percentages, QoL scores, qualitative themes) are summarized in Table 4 and detailed in text sections 3.2 to 3.5.
Results of syntheses	20a	For each synthesis, briefly summarise the characteristics and risk of bias among contributing studies.	Results, Sections 3.1–3.5, Pages 9–14<br>Summarized in text; synthesis draws on 3 qualitative, 3 quantitative descriptive, and 1 mixed-methods study.
	20b	Present results of all statistical syntheses conducted. If meta-analysis was done, present for each the summary estimate and its precision (e.g. confidence/credible interval) and measures of statistical heterogeneity. If comparing groups, describe the direction of the effect.	**N/A.** No statistical meta-analysis was conducted. Quantitative results are presented narratively and in descriptive ranges.
	20c	Present results of all investigations of possible causes of heterogeneity among study results.	Results, Section 3.1 & 3.5, Pages 9–10, 13–14<br>Attributes differences to variable primary tumor types (melanoma vs. NSCLC vs. sarcoma), age groups (AYAs vs. older adults), and differing psychometric tools.
	20d	Present results of all sensitivity analyses conducted to assess the robustness of the synthesized results.	**N/A.** No sensitivity analyses were performed.
Reporting biases	21	Present assessments of risk of bias due to missing results (arising from reporting biases) for each synthesis assessed.	Discussion, Section 4.1, Pages 14–15<br>Noted in limitations: 11 retrieved reports could not be obtained in full text during screening, potentially introducing selective reporting risk.
Certainty of evidence	22	Present assessments of certainty (or confidence) in the body of evidence for each outcome assessed.	Discussion, Section 4 & 4.1, Pages 14–15<br>Outlined narratively; confidence is limited by small total sample size ($n \approx 288$), cross-sectional designs, and geographical concentration in high-income countries.
**DISCUSSION**			
Discussion	23a	Provide a general interpretation of the results in the context of other evidence.	Discussion, Section 4, Pages 14–15<br>Interpreted through theoretical frameworks: Biographical Disruption Theory (Bury), Uncertainty in Illness Theory (Mishel), and existing cancer survivorship literature.
	23b	Discuss any limitations of the evidence included in the review.	Discussion, Section 4.1, Pages 14–15<br>Details sample heterogeneity, small sizes ($n=5$ to 90), geographic/melanoma bias (57.1%), and lack of longitudinal data.
	23c	Discuss any limitations of the review processes used.	Discussion, Section 4.1, Page 15<br>Highlights small total number of included studies ($n=7$), 11 unretrieved articles, and inability to perform meta-analysis due to heterogeneity.

PRISMA 2020 Checklist

Section and Topic	Item #	Checklist item	Location where item is reported
	23d	Discuss implications of the results for practice, policy, and future research.	Discussion, Section 4.2 & Conclusions, Pages 15–16<br>Recommends establishing transitional care protocols, specialized psycho-oncological screening, and prospective longitudinal trials.
**OTHER INFORMATION**			
Registration and protocol	24a	Provide registration information for the review, including register name and registration number, or state that the review was not registered.	Materials and Methods, Section 2, Page 3<br>Preregistered on Open Science Framework (OSF) portal (DOI: https://doi.org/10.17605/OSF.IO/NHETV).
	24b	Indicate where the review protocol can be accessed, or state that a protocol was not prepared.	Materials and Methods, Section 2 & Data Availability Statement, Pages 3, 16<br>OSF link provided in Section 2 and Data Availability Statement.
	24c	Describe and explain any amendments to information provided at registration or in the protocol.	**No reported.** No explicit protocol amendments or deviations are mentioned in the manuscript text.
Support	25	Describe sources of financial or non-financial support for the review, and the role of the funders or sponsors in the review.	Declarations (Funding), Page 16<br>Funded by Universidad Estatal de Santa Elena, grant number PSIC_01.
Competing interests	26	Declare any competing interests of review authors.	Declarations (Conflicts of Interest), Page 16<br>The authors declare no conflicts of interest.
Availability of data, code and other materials	27	Report which of the following are publicly available and where they can be found: template data collection forms; data extracted from included studies; data used for all analyses; analytic code; any other materials used in the review.	Declarations (Data Availability Statement & Supplementary Materials), Page 16<br>Dataset and PRISMA matrix are deposited on OSF (https://doi.org/10.17605/OSF.IO/NHETV). Supplementary PRISMA tables S1 and S2 available with publication.

From: Page MJ, McKenzie JE, Bossuyt PM, Boutron I, Hoffmann TC, Mulrow CD, et al. The PRISMA 2020 statement: an updated guideline for reporting systematic reviews. BMJ 2021;372:n71. doi: 10.1136/bmj.n71. This work is licensed under CC BY 4.0. To view a copy of this license, visit https://creativecommons.org/licenses/by/4.0/
