# Supplementary material for: The Psychosocial Burden of the ‘Lazarus Effect’: A Systematic Review of Survivor Guilt, Identity Shift, and Discontinuation Anxiety in Exceptional Responders to Cancer Immunotherapy
Source: Diseases. 2026 Sep 25;14(10):354. doi: 10.3390/diseases14100354 (PMC13703520; doi:10.3390/diseases14100354)
Supplement: Supplementary file 1 [file diseases-14-00354-s001.zip › Table_S3_ Unretrieved_reports_and_retrieval_attempts.pdf]

Table S3. Unretrieved reports and retrieval attempts

No	Bibliographic Reference	Retrieval Efforts Attempted	Primary Reason for Non-Retrieval / Access Barrier
1.	de Vries, F. E., Hafkamp, E., Huisman, A., Kamps, S., Egeler, M., Hales, S., Rodin, G., van de Poll-Franse, L., & Kool, M. (2026). Managing Cancer and Living Meaningfully (CALM) for Patients With Metastatic Cancer and Treatment Response. Psycho-Oncology, e70450. https://doi.org/10.1002/pon.70450	Interlibrary loan (ILL); open repository search; author email.	Publisher paywall (Wiley); no institutional access or self-archived copy.
2.	Alnajar, M., Kim, N., Roark, E., Park, M. J., Rose, M., Ansari, N., Perkins, R., Keddington, A., Ellington, L., & Tay, D. L. (2026). Palliative Care Perceptions and Needs Among Total Cancer Care Patients Undergoing Immunotherapy: A Mixed Methods Study. *Journal of Hospice & Palliative Nursing*, 28(2), E83-E95. https://doi.org/10.1097/NJH.0000000000001108	ILL request; repository check; ResearchGate message to lead author.	Subscriber paywall (Wolters Kluwer); author unreachable.
3.	Alsuliman, T., Musiu, P., Marjanovic, Z., Stocker, N., Seytre, N.-A., Lauga, L., Matin-Rojas, R.-M., Polomeni, A., Martins, G., Aftisse, H., Dulery, R., Magro, L., Mohty, M., Garidi, R., & Yakoub-Agha, I. (2025). Quality of Life in Patients With Malignant Blood Disorders: A Comprehensive Approach. *Hématologie*, 31(3), 208-241. https://doi.org/10.1684/hma.2025.1888	Proxy portal query; author email request.	Subscriber paywall (Cairn Pro); lack of site license.
4.	McGregor, B., O'Donnell, P. H., Balar, A., Petrylak, D., Rosenberg, J., Yu, E. Y., Quinn, D. I., Heath, E. I., Campbell, M., Hepp, Z., McKay, C., Steinberg, J., Regnault, A., Mazerolle, F., & Galsky, M. D. (2022). Health-related Quality of Life of Patients with Locally Advanced or Metastatic Urothelial Cancer Treated with Enfortumab Vedotin after Platinum and PD-1/PD-L1 Inhibitor Therapy: Results from Cohort 1 of the Phase 2 EV-201 Clinical Trial. *European Urology*, 81(5), 515-522. https://doi.org/10.1016/j.eururo.2022.01.032	ILL document delivery; author contact attempt.	Commercial paywall (Elsevier); no institutional license.
5.	Kieran, M. W., Caron, H., Winther, J. F., Henderson, T. O., Haupt, R., Hjorth, L., Hudson, M. M., Kremer, L. C. M., van der Pal, H. J., et al. (2021). A global approach to long-term follow-up of targeted and immune based therapy in childhood and adolescence. *Pediatric Blood & Cancer*, 68(8), e29047. https://doi.org/10.1002/pbc.29047	ILL request; pediatric repo search; author contact.	Publisher paywall (Wiley); subscription limits.
6.	Rogiers, A., Leys, C., De Cremer, J., Awada, G., Schembri, A., Theuns, P., De Ridder, M., & Neyns, B. (2020). Health-related quality of life, emotional burden, and neurocognitive function in the first generation of metastatic melanoma survivors treated with pembrolizumab: a longitudinal pilot study. *Supportive Care in Cancer*, 28(7), 3267–3278. https://doi.org/10.1007/s00520-019-05168-3	Institutional portal query; preprint search; author inquiry.	Individual paywall; no open copy.

7.	Milne, D., Hyatt, A., Billett, A., Gough, K., & Krishnasamy, M. (2020). Exploring the Experiences of People Treated With Immunotherapies for Advanced Melanoma and Those Caring for Them: "Real-World" Data. *Cancer Nursing*, 43(2), E97-E104. https://doi.org/10.1097/NCC.0000000000000683	ILL request; health database search; author email.	Subscriber-only paywall (Wolters Kluwer).
8.	Rischin D, Khushalani NI, Schmults CD, et al. Integrated analysis of a phase 2 study of cemiplimab in advanced cutaneous squamous cell carcinoma: extended follow- up of outcomes and quality of life analysis. Journal for ImmunoTherapy of Cancer 2021;9:e002757. doi:10.1136/ jitc-2021-002757	DOI/URL resolution; Wayback Machine archive check.	Broken URL (HTTP 404 Error / Inactive publisher link).
9.	Patrinely, J. R., Jr, Young, A. C., Quach, H., Williams, G. R., Ye, F., Fan, R., Horn, L., Beckermann, K. E., Gillaspie, E. A., Sosman, J. A., Friedman, D. L., Moslehi, J. J., & Johnson, D. B. (2020). Survivorship in immune therapy: Assessing toxicities, body composition and health-related quality of life among long-term survivors treated with antibodies to programmed death-1 receptor and its ligand. *European journal of cancer (Oxford, England : 1990)*, *135*, 211–220. https://doi.org/10.1016/j.ejca.2020.05.005	DOI link resolution; cache recovery attempt.	Broken URL (HTTP 404 Error / Dead indexing link).
10.	Blaes, A. H., Ma, L., Zhang, Y., & Peterson, B. A. (2011). Quality of life appears similar between survivors of indolent and aggressive non-Hodgkin lymphoma. *Leukemia & lymphoma*, *52*(11), 2105–2110. https://doi.org/10.3109/10428194.2011.593270	Direct URL verification; domain redirection check.	Broken URL (HTTP 404 Error / Inactive repository page).
11.	Messina, G., Lissoni, P., Bartolacelli, E., Fumagalli, L., Brivio, F., Colombo, E., & Gardani, G. S. (2007). Efficacy of IL-2 Immunotherapy in Metastatic Renal Cell Carcinoma in Relation to the Psychic Profile as Evaluated Using the Rorschach Test. *Anticancer Research*, *27*(4C), 2985-2988.	DOI target verification; digital archive search.	Broken URL (HTTP 404 Error / Server landing error).
